# Comparing Use Patterns and Acceptability of Mobile Digital Devices Between Care Recipients and Caregivers

**DOI:** 10.7759/cureus.41832

**Published:** 2023-07-13

**Authors:** Miaolung Shih, Wei-Chen (Miso) Lee, Huey-Ming Tzeng, Hani Serag, Mukaila Raji

**Affiliations:** 1 Osher Lifelong Learning Institute, University of Texas Medical Branch at Galveston, Galveston, USA; 2 Department of Family Medicine, University of Texas Medical Branch at Galveston, Galveston, USA; 3 School of Nursing, University of Texas Medical Branch at Galveston, Galveston, USA; 4 Department of Internal Medicine - Endocrinology, University of Texas Medical Branch at Galveston, Galveston, USA; 5 Division of Geriatrics & Palliative Medicine, Department of Internal Medicine, University of Texas Medical Branch at Galveston, Galveston, USA; 6 Department of Preventive Medicine and Population Health, University of Texas Medical Branch at Galveston, Galveston, USA

**Keywords:** mobile technology, dementia, digital device, caregiver, aging, smartphone application

## Abstract

Background: The use of smartphones and other digital devices (such as tablets and smartwatches) is important for the aging population to enhance and optimize communications with caregivers, families, friends, and providers. It also provides a platform for app-based activities to promote mental, physical, spiritual, and social well-being and virtual social connectedness. We, therefore, examined types of digital devices and categories of smartphone functions used by caregivers and care recipients in comparison to those without any caregiving roles.

Method: The project team has developed a smartphone app based on Buddhist meditative practice principles for the enhancement of the physical, mental, cognitive, and emotional well-being of older adults and their caregivers and tested it in Galveston, Houston, and Dallas, TX. The study comprised a convenient sample of older adults, including members or volunteers of the International Buddhist Progress Society-Dallas (IBPS Dallas) and the University of Texas Medical Branch Osher Lifelong Learning Institute (UTMB OLLI). The survey focused on people who were 55 years and older (n = 219), with 177 valid responses (~80.8%) meeting the study's inclusion criteria. The survey collected data on (1) caregiving role, (2) demographic characteristics and caregiving concerns, and (3) types of devices, functions, and utilization. Descriptive analysis and logistic regression were used to describe and compare patterns of smartphone function/use by the different groups, i.e., caregivers, care recipients, and neither.

Results: All of our survey respondents were 55 years and older, and among them, 17.5% were caregivers, 9.1% were care recipients, and 73.4% did not have any role. The majority of the caregivers were females (80.6%), and the average age of their care recipients was 66 years. The care recipients in our sample reported that the average age of their caregiver is only 55 years. Around three-fourths of caregivers reported that they have an app related to health or they are willing to use a health-related app, 32% of them use smart home appliances, whereas only 16% of people who are neither caregivers nor care recipients use such apps. Approximately 42% of caregivers reported taking care of their parents or parents-in-law, and their major concerns are about maintaining their income, scheduling tasks, and updating their knowledge as needed to better care for their loved ones. People use texting or messaging the most. However, the second and third highest utilization are different. The “neither” group significantly spends more time checking email and watching TV; the care recipients spend more time reading and watching TV (sedentary activities); the caregiver group spends more time on phone calls and listening to music.

Conclusions: Findings of different patterns of digital device use exist between caregivers, care recipients, and the “neither” group, with 75% of caregivers using a digital device app related to health or reporting willingness to use a health-related app developed from our study. Our findings of their caregiving experiences might also inform the design of different intervention programs aimed at promoting mental, physical, and social well-being, improving quality of life while reducing disease/disability burden for older adults, and preventing burnout among caregivers.

## Introduction

Older adults aged 65 years and older comprise a fast-growing segment of the US population, with the US Census Bureau predicting the older population outnumbering children by 2034 [1]. Accompanied by the increase in the older population is an increase in the prevalence of multiple chronic conditions such as dementia, diabetes, and depression [2]. There is also an increase in the proportion of older adults experiencing various levels of frailty, disability, suboptimal social network, and loneliness [3,4]. The age-related increase in mental and physical health conditions along with suboptimal social support in the face of accumulating disability is associated with poor quality of life, increased healthcare use/cost, and increased risk of nursing home placement [3]. Approximately half of nursing home residents have Alzheimer’s disease or/and related dementia (ADRD), a leading cause of loss of independent living and premature death in the older population in the US. About 11% of Medicare beneficiaries have a diagnosis of ADRD, with Texas being one of the states with the highest prevalence [5]. A 2019 report shows that total Medicare costs for older adults with ADRD ($25,213) are about three times higher than those without Alzheimer’s disease ($7,750) [6]. These statistics underscore the need to design and implement patient-centered and culture-appropriate interventions to help older adults with chronic conditions like ADRD age in place with good quality of life for older adults and their caregivers [7].

Governments worldwide are dedicated to supporting efforts, mostly pharmacological and resource-intensive approaches, aimed at designing and implementing interventions to reduce loneliness, expand social network/support, improve quality of life, and allow aging-in-place for the aging populations. One of the potential nonpharmacological solutions to this problem is to use technologies such as smartphone applications (apps), electronic wearables, and other internet-enabled mobile digital devices [8,9]. Smartphones, for example, are owned by 61% of older adults, but more refinements are needed to make smartphones easier to operate for older adults with deteriorating cognitive functions and motor and sensory impairments [10-12]. Likewise, only eight out of 118 smartphone applications on the market meet the needs of family caregivers of people with ADRD in terms of content and usability [13]. To meet the complex needs of a growing aging population, it is critical to transform more evidence-based activities into patient-centered digital devices that can be used in communities and home environments [14,15].

The use of smartphones and other digital devices (e.g., Apple Watch and iPad) is important for the aging population to enhance and optimize communications with caregivers, families, friends, and providers. It also provides a platform for app-based activities to promote mental, physical, spiritual, and social well-being and virtual social connectedness [7,16]. Informal caregivers, for example, can also use smartphones to reach formal healthcare providers for scheduling and counseling, as well as to do remote monitoring of patients' well-being. To develop a smartphone app to enhance the well-being of aging populations, it is important to collect data from both older adults and their primary sources of assistance to know what digital devices they currently use, what works, and what needs to be done for better application/adaptation of the digital devices for older populations.

The present study was focused on types of digital devices and categories of smartphone functions used by caregivers and care recipients in comparison to those without any of the caring roles. All survey respondents are non-institutionalized, and findings of their technology utilization will thus enable us to design an app that meets their day-to-day needs as they live in the communities. Moreover, comparing the utilization between caregivers and care recipients will provide some understanding of challenges for usability and insight, via the lens of users, of the association of the caregiving role and use of technology. Having the group of older adults without any caregiving role as the reference is also insightful to predict their change of technology utilization once they either become a caregiver or care recipient. Findings about the type, pattern, and frequency of utilization and their caregiving experiences from our study can thus inform a better design of different intervention programs aimed at promoting mental, physical, and social well-being, improving quality of life while reducing disease/disability burden for older adults and preventing burnout among caregivers.

## Materials and methods

Data source

The project team has developed a smartphone app based on Buddhist meditative practice principles for the enhancement of the physical, mental, cognitive, and emotional well-being of older adults and their caregivers in Galveston, Houston, and Dallas, TX. This project was jointly supported and conducted by the International Buddhist Progress Society-Dallas (IBPS Dallas) and the University of Texas Medical Branch Osher Lifelong Learning Institute (UTMB OLLI). The IBPS Dallas is a division of Fo Guang Shan, whose funder is Master Hsing Yun (1927-2023). Both Fo Guang Shan and UTMB share the same vision of altruism and humanity. With such a vision, the content of this smartphone app was drawn from two books: Humble Table (ISBN: 978-1-932293-56-2) and Pearls of Wisdom (ISBN: 0-9717495-6-6) and adapted to fit American culture to provide peaceful and harmonious messages to future app users.

Our study aims to improve the design of our smartphone app that meets the diverse needs of older Americans. The study sample was divided into three roles (caregiver, care recipient, or neither) to reflect on their level of burden. Caregivers might experience the burden of caregiving whereas care recipients might experience the burden of diseases, and both roles have a more challenging life than the "neither" group. We thus collected baseline data from community-dwelling older adults to identify patterns of use and the amount of time older adults spend on a variety of smartphone functions as well as the types of digital devices owned by our target populations (people aged 55 years and above). The questionnaire used was approved by the Institutional Review Board at the University of Texas Medical Branch (#21-0338).

Study sample

The study comprised a convenient sample of older adults including members or volunteers of IBPS Dallas and UTMB OLLI. The survey focused on people who were 55 years and older in the year 2022 and did not need surrogates to answer survey questions for them. The survey was completely anonymous, and respondents were not given any financial incentives. From March to July 2022, the study received 219 responses. After removing 10 incomplete responses and 33 responses that were found to be made by people under 55 years, there were 177 valid responses (~80.8%) that met the study inclusion criteria and were used for the final analysis.

Measurements

The online survey collected information about respondents’ demographics, caregiving role, amount of time they spend on each of the 16 smartphone functions, and devices they own as below. It is critical to evaluate the types, patterns, and frequency by caregiving roles. However, we did not control the demographic in the multivariate model because we used a convenient sample that did not comply with the rule of normal distribution. The generalizability of the finding will thus be limited.

Caregiving Role

Based on their responses, all survey respondents were divided into three different roles: being a caregiver, recipient, or neither. A caregiver is defined as an individual who is the primary source of assistance or care for another family member. The study does not include formal, paid caregivers such as nursing home aids or home health assistants in this study. The study also excluded all survey respondents younger than 55 years despite their role of caregiving since this study sought to compare the use of smartphones in the same generation of individuals aged 55 years and above.

Demographics and Caregiving Concerns

The first part of the survey asked respondents about their gender, age, race, education level, religion, and number of languages they can speak. Respondents were also asked about whom they take care of or who takes care of them, the age of the care recipient or caregiver, and their concerns.

Types of Devices, Functions, and Utilization

The second part of the survey was focused on the types of devices they own (multiple choices) and the amount of time they spend on each type of function per day (in minutes). Many respondents had difficulty recalling or counting how much time they exactly used each smartphone's function. As a result, we transformed numeric responses for the utilization question to a categorical variable with five degrees, including 1 = zero minutes or never use, 2 = 1-10 minutes, 3 = 11-30 minutes, 4 = 31-60 minutes, and 5 = more than an hour (per day). This needs assessment can reflect whether people aged 55 years and above are interested in using any app for health; if yes, what type of device they can use. This needs assessment also reflects the top three functions that everyone uses and the different utilization habits by caregiving role.

Analysis

A descriptive analysis was conducted to demonstrate survey respondents’ demographics and caregiving characteristics. All continuous measures (e.g., age) were summarized with a sample mean and standard deviation, and all categorical measures (e.g., gender) were summarized with the proportion of the sample in each category. Bivariate analysis was conducted to assess the association of caregiving role with their device ownership using the chi-square test and association analysis with any cell less than five was performed with Fisher exact test. ANOVA with the Kruskal-Wallis test was used to compare how much time was spent on each smartphone function by three different groups. Because ownership and types of utilization are multiple choices rather than exclusive categories, they do not account for multiple comparisons. For multi-comparison of smartphone use, statistical significance was defined as p-value < 0.01. All analyses were done by STATA v.17 (StataCorp LLC, College Station, TX).

## Results

Table 1 demonstrates that among 177 respondents aged 55 years and older, 31 (17.5%) were caregivers, 16 (9%) were care recipients, and 130 (73.5%) were neither a care recipient nor a caregiver. The average age of care recipients was 65.2 years and they were taken care of by people aged 55.9 years (std. = 2.88), i.e., about 10 years younger than them. The average age of caregivers was similar (66.0 years old), but they were taking care of people aged 70.9 years (std. = 5.63). Care recipients could speak more than two languages compared to another two groups that cannot (p = 0.002). Given that most (93.7%) of the care recipients are Asian Americans, it is possible that they can speak more than one language because of their immigration status. There was no statistical difference in terms of their worries (p = 0.068). The biggest worries for both care recipients and caregivers were low income, busy schedules, and lack of caring knowledge. One-third of caregivers reported they are often worried about death and uncertainty in the health of the people they take care of.

**Table 1:** Characteristics of survey respondents

Total: 177	Neither (N = 130)	Care recipient (N = 16)	Caregiver (N = 31)	p-value
Gender (n, %)				0.390
Male (n = 49)	37 (28.5%)	6 (37.5%)	6 (19.4%)	
Female (n = 128)	93 (71.5%)	10 (62.5%)	25 (80.6%)	
Age (mean, std.)	65.6 (0.57)	65.2 (1.54)	66.0 (1.07)	0.860
Race (n, %)				0.031
African American (n = 5)	4 (3.1%)	0 (0.0%)	1 (3.2%)	
Asian American (n = 103)	67 (51.5%)	15 (93.7%)	21 (67.7%)	
Hispanic (n = 6)	4 (3.1%)	0 (0.0%)	2 (6.5%)	
White (n = 63)	55 (42.3%)	1 (6.3%)	7 (22.6%)	
Education (n, %)				0.759
Less than high school (n = 4)	3 (2.3%)	1 (6.3%)	0 (0.0%)	
High school or equivalent (n = 24)	20 (15.4%)	1 (6.3%)	3 (9.7%)	
Associate or bachelor’s degree (n = 71)	51 (39.2%)	7 (43.7%)	13 (41.9%)	
Graduate school or above (n = 78)	56 (43.1%)	7 (43.7%)	15 (48.4%)	
Religion (n, %)				0.006
Christian or Catholic (n = 54)	45 (34.6%)	1 (6.3%)	8 (25.8%)	
Buddhism (n = 76)	46 (35.4%)	13 (81.2%)	17 (54.8%)	
Others (n = 47)	39 (30.0%)	2 (12.5%)	6 (19.4%)	
Number of languages (mean, std.)	1.4 (0.05)	2.1 (0.17)	1.7 (0.11)	0.002
Age of people you take care or people take care of you (mean, std.)	-	55.9 (2.88)	70.9 (5.63)	<0.001
Relationship (n, %)				0.029
Kids or grandkids	-	4 (25.0%)	6 (19.4%)	
Spouse	-	7 (43.8%)	8 (25.8%)	
Parents or parents-in-law	-	0 (0.0%)	13 (41.9%)	
Other relatives	-	4 (25.0%)	4 (12.9%)	
Missed reporting	-	1 (6.2%)	0 (0.0%)	
What worry you a lot (multiple choices) (n, %)				0.068
Ability (e.g., income, knowledge, and schedule)	-	8 (50.0%)	13 (41.9%)	
Distance	-	4 (25.0%)	4 (12.9%)	
Their health or uncertainty of death	-	0 (0.0%)	10 (32.3%)	
Missed reporting	-	4 (25.0%)	4 (12.9%)	

Table 2 illustrates that more than 60% of respondents own a laptop or notebook across three groups. The caregiver group has the highest percentage of people owning a laptop (80.7% > neither: 65.4% > recipient: 62.5%) and smart home appliances (32.3% > neither: 16.2% > recipient: 12.5%). Additionally, when asked if they have any app related to health management, more caregivers either already have one (48.4% > recipient: 43.8% > neither: 38.9%) or like to have one (25.8% > neither: 15.9% > recipient: 12.5%).

**Table 2:** Comparing interests in using technologies for health by their roles

n (%)	Neither (130, 100.0%)	Care recipient (16, 100.0%)	Caregiver (31, 100.0%)	p-value
Use any app for health				0.342
No, I don’t want one	57 (45.2%)	7 (43.8%)	8 (25.8%)	
No, but I like to try	20 (15.9%)	2 (12.5%)	8 (25.8%)	
Yes	49 (38.9%)	7 (43.8%)	15 (48.4%)	
Own any device (multiple choices)				
Tablet	63 (48.5%)	5 (31.3%)	13 (41.9%)	0.424
Laptop/notebook	85 (65.4%)	10 (62.5%)	25 (80.7%)	0.235
Desktop	42 (32.3%)	7 (43.8%)	11 (35.5%)	0.646
Smartwatch	36 (27.7%)	3 (18.8%)	8 (25.8%)	0.743
Smart home appliance	21 (16.2%)	2 (12.5%)	10 (32.3%)	0.095
None of them	13 (10.0%)	0 (0.0%)	0 (0.0%)	0.079

Table 3 shows the different degrees of utilization by each of the three groups. Of people aged 55 years and older, the function they use the most is texting or sending messages. For the “neither” group, they also use smartphones more for email (score = 3.00) and watching TV or movie (score = 2.81). The care recipients use smartphones for sedentary activities like reading (score = 3.63) and watching TV or movie (score = 3.56) for more than 30 minutes a day. Finally, the caregivers report that they use smartphones for phone calls (score = 3.42) and email (score = 3.23) for more than 30 minutes a day. When comparing the three groups’ degrees of utilization, caregivers have the highest use of smartphones for phone calls whereas care recipients have the highest use for reading and attending online classes.

**Table 3:** Comparing smartphone utilization by their roles Note: The responses are on a five-point Likert scale from 1 = zero minutes or never use, 2 = 1-10 minutes, 3 = 11-30 minutes, 4 = 31-60 minutes, to 5 = more than an hour (per day). *: p < 0.01.

	Neither/Ref (mean, std.)	Care recipient (mean, std.)	Caregiver (mean, std.)
Texting or messaging	3.34 (0.11)	3.81 (0.29)	3.52 (0.18)
Phone calls	2.79 (0.09)	3.13 (0.34)	3.42 (0.15)*
Email	3.00 (0.10)	3.19 (0.34)	3.23 (0.22)
Social media (e.g., Facebook)	2.75 (0.12)	3.19 (0.28)	3.19 (0.21)
Music or podcasts	2.39 (0.12)	2.63 (0.26)	3.03 (0.26)
Play games	2.08 (0.13)	2.06 (0.32)	1.65 (0.21)
Watch TV or movie	2.81 (0.14)	3.56 (0.35)	2.52 (0.30)
Shopping, ordering	2.18 (0.09)	1.88 (0.20)	2.19 (0.16)
Reading, attending online classes	2.58 (0.12)	3.63 (0.34)*	2.68 (0.22)
Web surfing (e.g., find a restaurant)	2.50 (0.09)	2.44 (0.20)	2.55 (0.16)
Map, direction	2.32 (0.07)	2.75 (0.27)	2.61 (0.18)
Calendar, note taking	2.25 (0.08)	2.63 (0.36)	2.52 (0.16)
Banking or financing	2.05 (0.08)	2.06 (0.23)	2.26 (0.16)
Take/edit photos or videos	2.21 (0.07)	2.13 (0.24)	2.48 (0.16)
Weather, news	2.54 (0.08)	2.75 (0.23)	2.65 (0.16)

## Discussion

The growing aging population with an increased prevalence of multiple chronic conditions leads to increasing demand for services by caregivers. The study recruited a convenient sample through our collaborators in three different cities. All of our survey respondents were aged 55 years and older and among them, 17.5% play the role of caregiver, which is slightly smaller than the percentage in Edwards’ national representative sample that shows 20% of people older than 65 years are caregivers [17]. Similar to the national representative sample, the majority of the caregivers in our sample are female, and the average age of the people they take care of is 70 years [17,18]. However, the care recipients in our sample also reported that the average age of their caregiver is only 55 years. Because people aged 55 years are likely to still be in the workforce, more strategies to alleviate the burden of caregiving, enhance their mental and physical health, and enable the work-life balance would be imperative.

Transforming healthcare delivery through advancing technology to meet the complex needs of the aging population has been warranted [8]. This study compared the ownership and types of digital technologies and the degree, intensity, and patterns of utilization of smartphone applications between caregivers and care recipients. The study of the pattern of digital device use between caregivers and care recipients will inform the development of a more user-friendly and patient-centered smartphone app to improve emotional and cognitive functions and social interconnectedness in older adults, especially when in-person day-to-day interaction is limited, as was the case during the coronavirus disease 2019 (COVID-19) lockdown. Around three-fourths of caregivers in our sample reported that they have an app related to health or they are willing to use a health-related app. Also, 32% of them use smart home appliances, whereas only 16% of people who are neither caregivers nor care recipients use them. Although both findings are not statistically significant, it provides technology developers the opportunity to design a more specific app or program to help caregivers with the challenges they face [19]. Additionally, approximately 42% of caregivers reported taking care of their parents or parents-in-law, and their major concerns are about maintaining their income, scheduling tasks, and updating their knowledge as needed to better care for their loved ones. A well-designed app can help with these concerns, especially the latter two concerns. Designing an app or a smart home appliance that shares caregivers' responsibilities/needs and improves their knowledge of taking care of parents has the potential to meet their caregiving needs, lower caregiver burden, and help the care recipients age in place.

Low technology literacy or design barriers have disproportionately affected older adults [20,21]. In addition, social withdrawal has been identified as one common issue among older adults and the COVID-19 pandemic has worsened the situation [22]. Among 15 functions, the result showed that people use texting or messaging the most, which does not require much of learning a new skill or muscle movement. However, the second and third highest utilization are different and reflect different lifestyles by different caregiving roles. The “neither” group spends more time checking their emails and watching TV; the care recipients spend more time reading and watching TV; the caregiver group spends more time on phone calls and listening to music. Our findings may be explained by their health status and degree of function that shape their lifestyle differently but also by the COVID-19 context during the study period. Different utilization patterns may inform healthcare providers, technology developers, and policymakers to prioritize resources to approach different populations while taking the environment into consideration.

We recognize that the study has several study limitations, and the findings should be interpreted with caution. First, the study only drew a convenient sample (N = 177) within a limited period of time (October to December 2022). Because more than 55% of survey respondents are Asian Americans, the finding may be more relevant to this particular population in the caregiving role. Second, the survey did not ask participants’ concerns about using technologies such as cost, privacy, and confidentiality. Although the study finding may provide indications for future technology design and marketplace, it is important to further investigate caregivers’ and care recipients’ concerns and negative perceptions about technologies. Third, the study used a five-point Likert scale to investigate the length of time older adults spent on each smartphone function. Additionally, the result was drawn from the self-reported surveys, and several respondents had difficulty remembering their actual frequency of use. The result should be interpreted by the definition of each category rather than by the absolute score number, such as a score of 3 means 11-30 minutes, which is way longer than a score of 2, which means 1-10 minutes.

## Conclusions

The study is focused on people aged 55 years and older who are likely to become a caregiver or a care recipient soon in their lives. They are also at a higher risk of developing cognitive impairments, with care recipients reporting concerns and potential struggles with decision-making, daily activity performing, and even medication management. With the goal of disease prevention and control, a better understanding of their smartphone utilization and a person-centered development of an app has huge potential to enhance caregivers’ and care recipients’ cognitive functions, emotional well-being, and social connections. More large-sample quantitative and qualitative studies, especially in more racially and ethnically diverse populations, to identify nonpharmacological strategies for alleviation of caregiving burden, social isolation, and loneliness in older adults are urgently needed.
